# The effect of Vagus nerve stimulation (VNS) on seizure control, cognitive function, and quality of life in individuals with drug‐resistant epilepsy: A systematic review article

**DOI:** 10.1002/epi4.13066

**Published:** 2024-10-30

**Authors:** Daniel Molla Melese, Abebaye Aragaw, Wondyefraw Mekonen

**Affiliations:** ^1^ Department of Biomedical Science, Asrat Woldeyes Health Science Campus Debre Berhan University Debre Berhan Ethiopia; ^2^ Department of Physiology, College of Health Sciences Addis Ababa University Addis Ababa Ethiopia

**Keywords:** drug‐resistant epilepsy, Ethiopia, quality of life, Vagus nerve stimulation

## Abstract

**Objectives:**

To evaluate the effect of vagus nerve stimulation (VNS) on seizure control, cognitive functions, and quality of life in individuals with drug‐resistant epilepsy.

**Methods:**

An extensive search of electronic databases was carried out in order to carry out this systematic review. The databases Google Scholar, Embase, PubMed, and the Cochrane Library were searched first to carryout gray literature. To reduce the quantity of pointless studies in the advanced search, the search is limited to “human studies” and “English language” publications only. Combining keywords and Medical Subject Headings (MeSH) terms like (“Vagus Nerve Stimulation” OR “VNS”) AND (“Epilepsy” OR “Seizure Control”) AND (“Cognitive Function” OR “Quality of Life”). Studies that have been published up to November 30/2023 were included.

**Results:**

The search strategy yielded a total of 392 relevant studies. The mean age of participant's ranges from 11 years to 33 years. The duration of follow‐up ranging from 6 to 36 months. Eleven studies were included in the review. The mean≥50% response rate after VNS therapy was 56.94% ranged from 48.90% to 83.00%. Four and three studies provided information about Quality of Life in Epilepsy Inventory (QOLIE‐31) and The Liverpool Seizure Severity Scale (LSSS) questionnaires respectively.

**Significance:**

Epilepsy is a chronic disease characterized by sudden abnormal discharge of brain neurons, which leads to transient brain dysfunction and the presence of spontaneous recurrent seizures. Vagus nerve stimulation has recently been proposed as a potential tool in the treatment of seizure, depressive symptoms, and cognitive impairments. There has been variation in the effects of VNS treatment on seizure control, cognitive functions, and quality of life among patients with drug‐resistant epilepsy. So, a comprehensive review of exciting literature is important to see the pooled effect. Previous systematic review and meta‐analysis papers were mostly randomized control trial type with specific diseases. The use of a wider variety of study designs than only randomized controlled trials is important. So, we included retrospective and prospective cohort studies in addition to randomized control trials. This enables a more thorough assessment of the connection between quality of life, cognitive function, and vagus nerve stimulation. In addition, the paper looks at a wide range of disease kinds and patterns. We have established a uniform and comprehensive approach throughout the selected studies by mandating the inclusion of all three crucial parameters: vagus nerve stimulation, cognitive function, and quality of life.

**Plain Language Summary:**

This systematic review examined 392 relevant studies on vagus nerve stimulation (VNS) therapy, with participants ranging from 11 to 33 years old and follow‐up durations of 6–36 months. Eleven studies were included, and the mean response rate after VNS therapy was 56.94%, ranging from 48.90% to 83.00%. The review also reported on quality of life and cognitive function, and seizure severity frequency result from several studies.

**Key points**

1. The search strategy yielded a total of 392 relevant studies.
2. Among this, 11 studies which fulfill the inclusion criteria were included.
3. The age of the participant ranges from 11 years to 33 years.
4. The mean >50% response rate after VNS therapy was 56.94%.
5. Vagus nerve stimulation improve seizure, quality of life, and cognitive function.

## 1 INTRODUCTION

Sudden aberrant discharge of brain neurons, resulting in temporary brain malfunction and the occurrence of spontaneous recurrent seizures, is the hallmark of epilepsy, a chronic condition. Worldwide estimates place the lifetime prevalence of epilepsy between 1% and 5%.^1^ Drug‐resistant epilepsy (DRE) is defined by the International League against Epilepsy (ILAE) as an uncontrollable seizure within two anti‐seizure medicines (ASM) that have been carefully chosen and well tolerated, regardless of whether they are used in combination or as monotherapy.^2^

### 1.1 Treatment of epilepsy

Drugs, surgery, neuromodulation, and a ketogenic diet are used to treat epilepsy. One option for treating epilepsy is surgical resection, although not every patient is a good candidate for this operation. Despite surgically removing the epileptogenic center, 30%–40% of patients still experience seizures.^3^ Therefore, there should be a different method for epilepsy sufferers to lessen their epilepsy symptoms and suffering in addition to medication and surgery.

**FIGURE 1:**
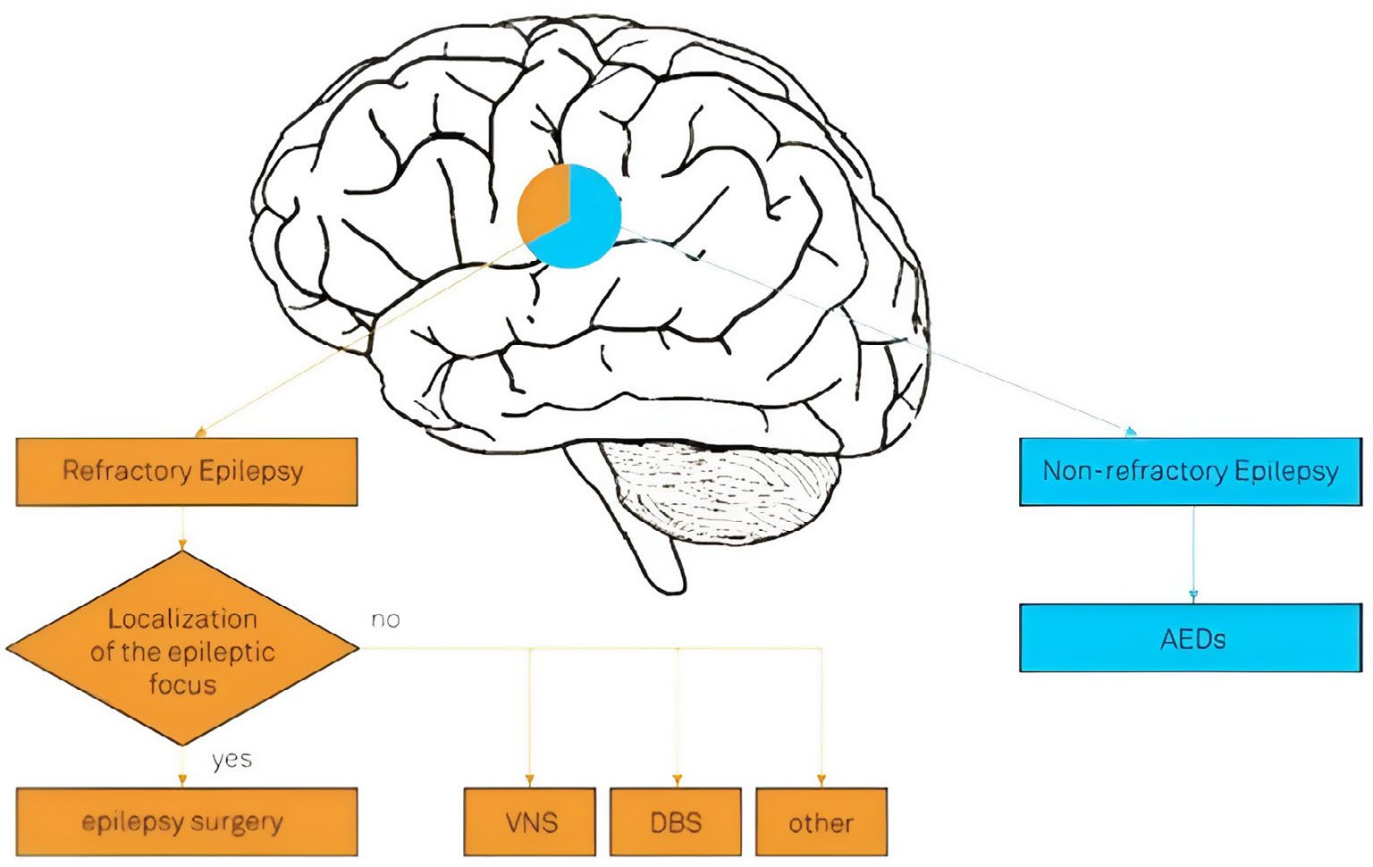
Diagram of therapy options in epilepsy management.^6^

Electrical vagus nerve stimulation (EVNS) is one palliative treatment available to patients who are not eligible for reconstructive surgery. Its exact mode of action is unknown, however, it may entail widespread cortical and subcortical cerebral metabolic alterations through brainstem and solitary tract nucleus activity modulation.^4^ Its effectiveness is associated with a decrease in seizure frequency and duration as well as an enhancement in life quality^5^ (Figure 1).

### 1.2 Mechanism of action of vagus nerve stimulation

Therapy‐resistant epilepsy patients can benefit from supplementary vagus nerve stimulation (VNS) treatments. Most of the time, it is still unclear how VNS works. One way that VNS works is by way of the tryptophan metabolism route.

### 1.3 Tryptophan metabolism

One of the necessary amino acids, tryptophan is mostly found in diet. Despite the fact that certain bacterial species like E. coli have been found to generate tryptophan.^7^

Beyond its function in protein biosynthesis, tryptophan in mammals can be converted via two primary pathways: the first is the serotonin pathway, which produces serotonin and melatonin, and the second is the kynurenine pathway, which produces kynurenine and its byproducts.^8^

### 1.4 Serotonin pathway

Tryptophan hydroxylase (TPH; two variants: TPH1 and TPH2) is the rate‐limiting enzyme in the serotonin pathway, converting tryptophan down the route. Tryptophan decarboxylase 9 then proceeds to further convert the resultant product, 5‐hydroxytryptophan (5‐HTP), to serotonin (or 5‐hydroxytryptamine, 5‐HT).^9^

### 1.5 Kynurenine pathway

Indoleamine 2, 3‐dioxygenase (IDO1 and IDO2) converts tryptophan to N‐formyl kynurenine, which is then transformed to kynurenine.^10^ This process starts the kynurenine pathway.

After kynurenine is produced, the metabolism of kynurenine divides into two main branches. Kynurenine aminotransferases (KATs) catalyze the transamination of kynurenine along the first branch, resulting in the formation of kynurenic acid (KYNA).^11^ On the second branch, kynurenine is transformed into 3‐hydroxykynurenine (3‐HK) and then into quinolinic acid (QUIN), which is started by the enzyme kynurenine‐3‐monooxygenase (KMO).^11^ Microglia cells are the primary makers of QUIN, whereas astrocytes are the primary producers of KYNA, mostly expressing KAT II.^12^

**FIGURE 2:**
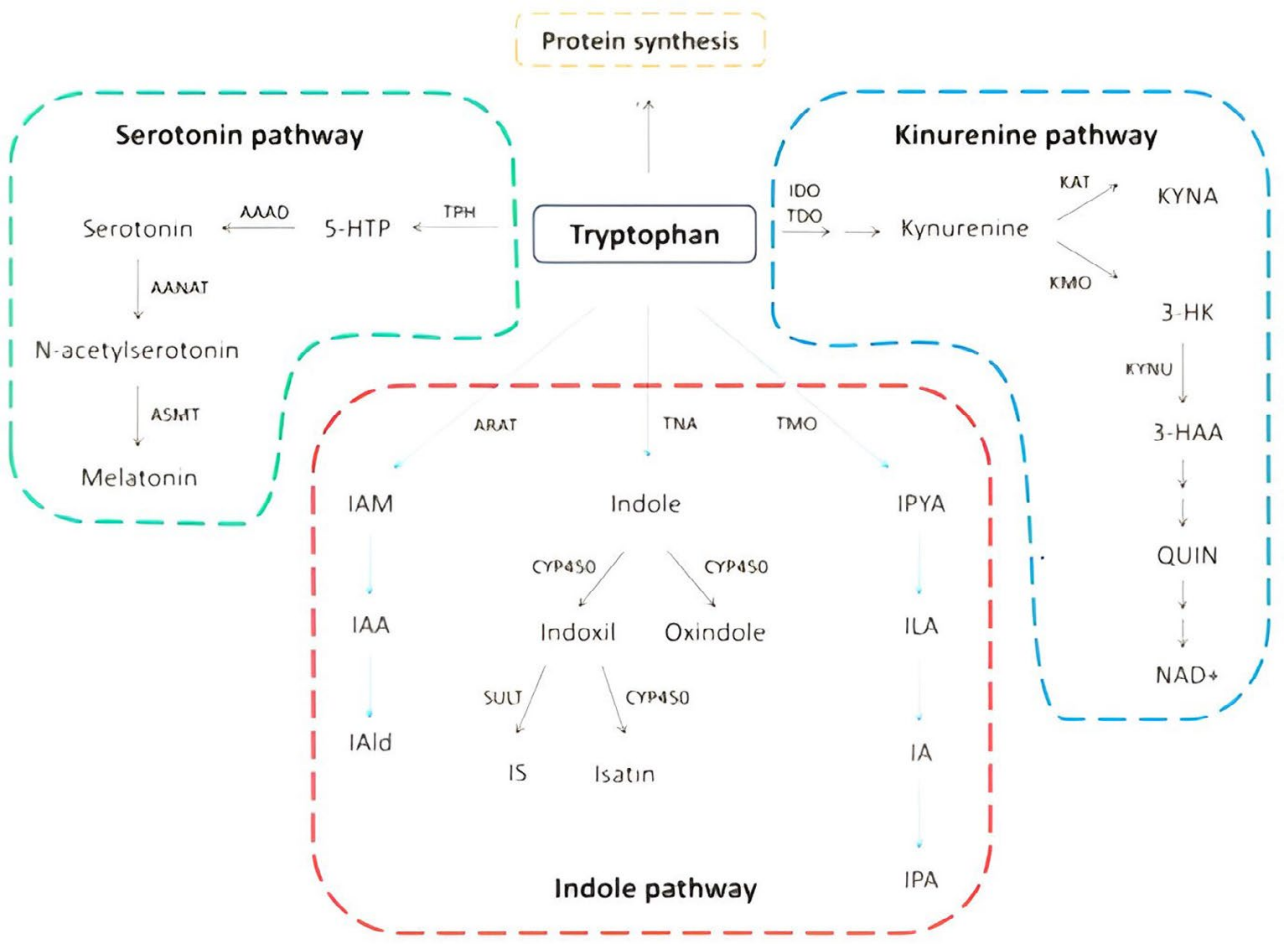
Schematic overview of tryptophan metabolic pathways.^14^

The two main neuroactive kynurenine catabolites are QUIN and KYNA. The N‐methyl‐Daspartate (NMDA) receptor is a type of glutamate receptor that is important for controlling synaptic plasticity in the brain. However, excessive activation of this receptor can be harmful to the nervous system. While QUIN functions as an NMDA receptor agonist and has neurotoxic effects, KYNA operates as an NMDA receptor antagonist and has a neuroprotective impact^13^ (Figure 2).

Furthermore, VNS has lately been suggested as a possible therapy option for major depressive disorders (MDD).^15^ Nonetheless, there has been variability in research regarding how VNS treatment affects patients with drug‐resistant epilepsy (DRE) in terms of seizure control, cognitive function, and quality of life (QOL).

## 2 METHODOLOGY

### 2.1 Study design

In order to find out how VNS affected patients with DRE's quality of life, cognitive abilities, and seizure outcomes, a systematic review was carried out. This work adheres to the Preferred Reporting Items for Systematic Reviews and Meta‐Analysis (PRISMA)^16^ established protocols for systematic reviews and meta‐analyses. Following the PRISMA recommendations, the research question as well as the inclusion and exclusion criteria were prepared. The PRISMA flow diagram was used to illustrate the processes for the screening and selection of relevant studies.

### 2.2 Searching strategies

An extensive search of electronic databases was carried out in order to carry out this systematic review. Finding both published and unpublished studies was the goal of the search approach. The databases Google Scholar, Embase, PubMed, and the Cochrane Library were searched first to carryout gray literature. The reference lists of the studies that were obtained were examined by getting in touch with pertinent specialists and visiting the organization's website in order to get papers that are not available through search engines and databases.

To reduce the quantity of pointless studies in the advanced search, the search is limited to “human studies” and “English language” publications only. Combining keywords and Medical Subject Headings (MeSH) terms like (“Vagus Nerve Stimulation” OR “VNS”) AND (“Epilepsy” OR “Seizure Control”) AND (“Cognitive Function” OR “Quality of Life”). Search Date Range: From the inception of publication on each database up to November 30/2023. Studies that have been published up to November 30/2023 were included in the initial search. The PRISMA guidelines were followed throughout the study selection process and reporting of the systematic review results.

#### 2.2.1 Study selection

The Articles gathered from different sources was exported to Endnote 20, and duplicates was identified and removed. The remaining articles was assessed with respect to the topic, study area, study participants, and language.

#### 2.2.2 The verbal fluency test (VFT)

VFT were used to assess memory and language function. The Quality of Life in Epilepsy Inventory **(QOLIE‐31)** and The Liverpool Seizure Severity Scale **(LSSS)** tool were used to measure the perception of seizure severity with drug resistance epilepsy.

### 2.3 Eligibility criteria

#### 2.3.1 Inclusion criteria

1. Children's ≥4 years old and adults who have drug resistance epilepsy
2. Duration of follow‐up ≥5 months and above were included
3. Cohort studies and randomized, double‐blind, placebo‐controlled trials study designs were used.
4. Population: A clinical diagnosis of drug‐resistant epilepsy was required for study participants.
5. Interventions: Research in which the VNS device was implanted in every subject.
6. Outcomes: Patients who showed at least a 50% reduction in the frequency of their seizures were classified as good responders to VNS therapy, whereas those who showed less than a 50% decrease were classified as poor responders.

#### 2.3.2 Exclusion criteria

1. Reviews, case reports, and studies using animals were not included.

2. If there was no mention of a definitive result, the study was disregarded.

3. The patient characteristics were not disclosed in the study.

### 2.4 Data extraction

Once the eligible studies were identified, the data were extracted using a Microsoft Excel spreadsheet format that was adapted from the JBI data extraction format. This format included the following: (1) general information, such as the year of publication and the first author; (2) study characteristics, such as the number of subjects, sex ratio (male/female), mean subject age, percentage of responders, and duration of follow‐up; and (3) epilepsy duration, seizure type, quality of life, and cognitive function.

## 3 RESULTS

**FIGURE 3:**
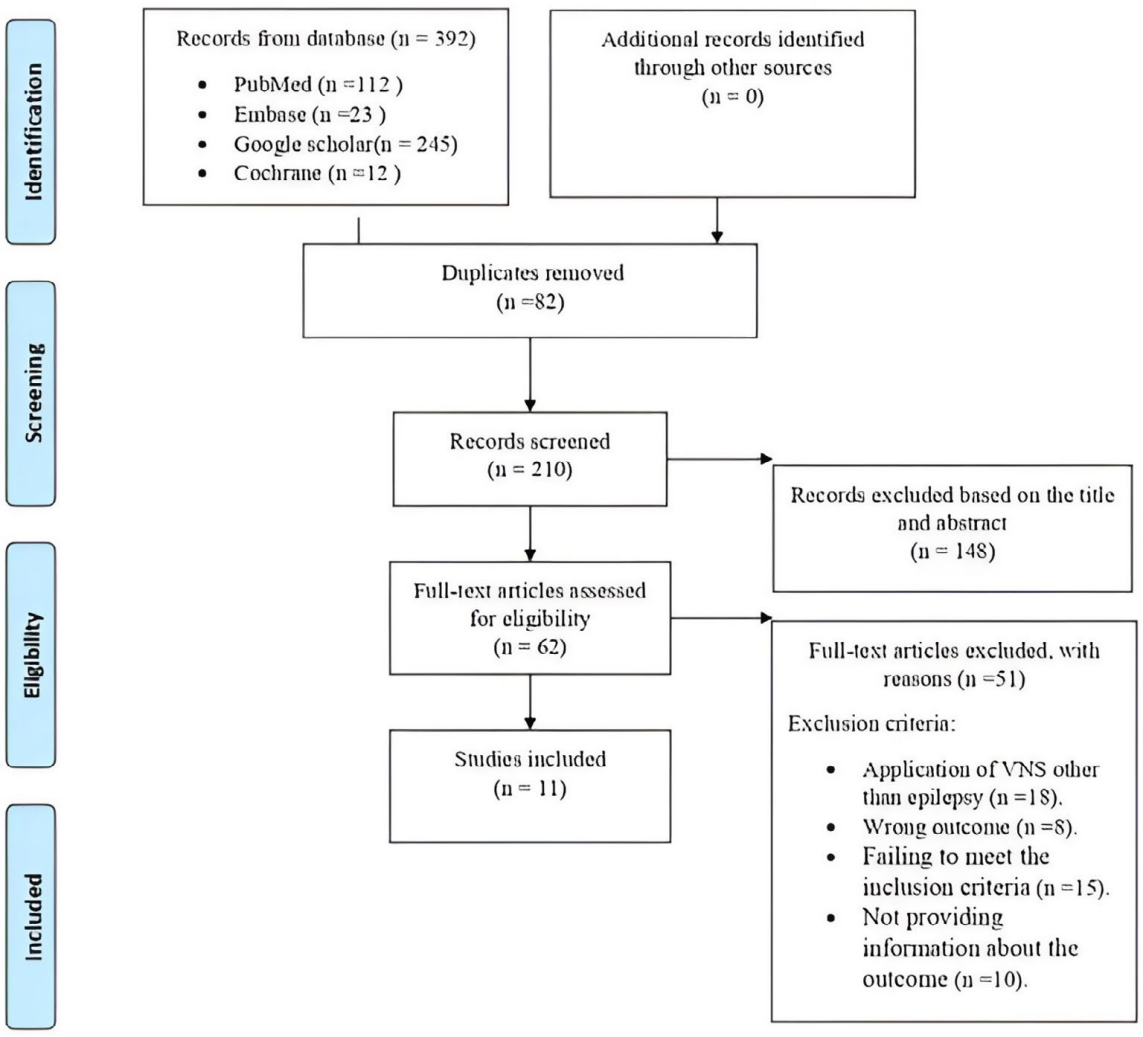
PRISMA 2020 Flow Diagram.

A thorough overview of the body of research on the impact of VNS in people with drug‐resistant epilepsy was found in the systematic review. A total of 392 pertinent studies that satisfied the inclusion criteria were found using the search technique. The studies used a variety of designs, including cohort studies and randomized controlled trials. Findings from Cochrane (*n* = 12), Embase (*n* = 23), Google Scholar (*n* = 245), and PubMed (*n* = 112). After removing 82 duplicates, 210 studies remained, of which 148 were disqualified due to either non‐English text in the abstract or title. The remaining 62 pertinent papers underwent a full‐text evaluation to determine their eligibility. Eighteen studies were removed from the full‐text evaluation for using VNSs for purposes other than epilepsy, fifteen for not meeting the research type inclusion requirements, ten for not giving information about the outcome, and eight studies for incorrect outcome, others are correct. The evaluation comprised eleven studies: two randomized controlled trials (RCT),^17^, ^18^ four prospective studies (PS)^19^, ^20^, ^21^, ^22^ two retrospective studies (RS)^23^, ^24^ and three experimental studies (Expr).^25^, ^26^, ^27^ The first database search produced eleven of them (Figure 3).

### 3.1 Sociodemographic and clinical characteristics

The systematic review offers a worldwide perspective on VNS in the treatment of epilepsy by incorporating studies from Korea,^25^ Croatia,^28^ Italy,^19^ Netherland,^20^ China,^23^ Japan,^21^ Saudi Arabia,^24^ Europe and Canada,^18^ and Romania.^22^ A varied body of data is produced by various study designs, such as randomized long‐term trials,^18^ experimental trials (Hajnšek et al., 2011), prospective cohorts,^25^ and retrospective cohorts.^24^

The participants' ages range from 11 to 33 years old on average, which reflects the wide age variation observed in the investigations. There is a significant range in the follow‐up duration, from 6 months to 36 months, which suggests different timelines for assessing the effects of VNS. A range of epilepsy forms are taken into consideration, including multifocal,^25^ CPS (Complex Partial Seizures) (Hajnšek et al., 2011), CPS+ aura,^20^ generalized,^23^ tonic–clonic seizure,^21^ myoclonic,^24^ and GTC (Generalized Tonic–clonic),^27^ demonstrating the versatility of VNS in treating epilepsy. Numerous outcomes are reported in the research, including the frequency of seizures both before and after VNS treatment. The 50% response rate following VNS ranges from 48.90% to 83.00%, demonstrating the broad applicability of VNS in lowering seizure frequency (Table 1).

**TABLE 1:** Sociodemographic and clinical characteristics of the study.

Authors	Year	Location	Study type	Sample size	Type of epilepsy	Mean age	Mean duration of follow‐up	Mean duration of disease	Seizure frequency at baseline	Seizure frequency after VNS	50% response rate
(Kim et al.^25^)	2021	Korean	Prospective cohort	25	Multifocal	30.4	13.7 months	10 years	8.18/week	2.5/week	50%
(Kim et al.^26^)	2022	Korean	Prospective cohort	24	Multifocal	30.6	11 months	20.2 years	Unknown	Unknown	83.00%
(Hajnšek et al.^28^)	2011	Croatia	Experimental trial	11	CPS	26.62	6 years	19.34 years	16/month	8/month	51.67%
(Colicchio et al.^19^)	2012	Italy	Prospective cohort	53	Unknown	30.67	55.96 months	22.28 years	30.78/month	13.3/month	51%
(Ardesch et al.^20^)	2007	Netherlands	Prospective cohort	19	CPS + aura	33	4 years	23 years	28/month	14/month	50%
(Shan et al.^23^)	2022	China	Retrospective cohort	45	Generalized	27.9	32.5 months	11.8 years	Unknown	Unknown	48.90%
(Kawai et al.^21^)	2017	Japan	Prospective cohort	362	tonic–clonic seizure	23	36 months	15.6 years	106/week	62/week	58.80%
(Bamogaddam et al.^24^)	2020	Saudi Arabia	Retrospective cohort	18	Myoclonic, GTC	20.33 years	36 months	19.61 years	Unknown	Unknown	54%
(Ryvlin et al.^18^)	2014	Europe and Canada	Randomized Long‐term trial	112	focal epilepsy	Unknown	12 months	Unknown	Unknown	Unknown	50%
(Tohanean et al.^22^)	2018	Romania	Prospective cohort	28	Unknown	32.9	6 months	20.7 years	49/month	10/month	64%
(Hallböök et al.^27^)	2005	Sweden	Prospective longitudinal study	15	complex partial	11	9 months	8.5 years	51/month	18/month	65%

### 3.2 Quality of life

Numerous research investigations have shown favorable results in relation to the amelioration of depressive symptoms, mood, overall quality of life, and quality of life ratings among epileptic patients. According to Kim et al.^25^ 84% of patients had less depressed symptoms. All patients showed improvements in mood and overall quality of life, according to Hajnšek et al. (2011). The QOLIE‐31 scores improved by 54.0%, according to Shan et al.^23^ QOL scores showed a significant 54.7% improvement, according to Kawai et al.^21^ By utilizing the Quality of Life in Epilepsy Inventory‐89 overall score, Ryvlin et al.^18^ discovered a higher improvement in HRQoL.

Nevertheless, a different study conducted by Tohanean et al.^22^ found that while VNS may lessen seizures, it does not always have an effect on the patient's quality of life. Finally, an improvement in the parents' perception of quality of life was seen by Hallböök et al.^27^ in 80% of cases (Table 2).

**TABLE 2:** Assessment of quality of life of the studies.

Author	Year	Location	Mean age	Sample size	Study type	Type of epilepsy	Mean duration of follow‐up	Mean duration of disease	Mean seizure frequency	QOLIE‐31/LSSS score
Kim et al.^25^	2021	Korean	30.4	25	Prospective cohort	Multifocal	13.7 months	10 years	8.18/week	About 84% of patients showed reductions in depressive symptoms
Hajnšek et al., 2011	2011	Croatia	26.62	11	Experimental trial	CPS	6 years	19.34 years	16/month	Improvement of mood and the general quality of life in all patients
Shan et al.^23^	2022	China	27.9	45	Retrospective cohort	Generalized	32.5 months	11.8 years	Unknown	QOLIE‐31 scores improved (54.0%)
Kawai et al.^21^	2017	Japan	23	362	Prospective cohort	tonic–clonic seizure	36 months	15.6 years	106/week	QOL score markedly improved (54.7)
(Ryvlin et al.^18^)	2014	Europe and Canada	Unknown	112	Randomized Long‐term trial	focal epilepsy	12 months	Unknown	Unknown	Greater improvement in HRQoL (5.5) using Quality of Life in Epilepsy Inventory‐89 total score
(Tohanean et al.^22^)	2018	Romania	32.9	28	Prospective cohort	Unknown	6 months	20.7 years	49/month	The data show no correlation between the QOLIE‐31 final score and the seizure reduction rate.
(Hallböök et al.^27^)	2005	Sweden	11	15	prospective longitudinal study	complex partial	9 months	8.5 years	51/month	In this study, 80% of the children had an improvement in parent's conception of the child's QOL

### 3.3 Cognitive functions

According to research by Kawaki et al.^21^ no appreciable changes in cognitive impairment were seen. On the other hand, 77.8% of participants in the study by Oh et al.^29^ reported an improvement in cognitive impairment. There were no variations in cognitive functioning between the pre‐ and post‐vagus nerve stimulation (VNS) periods in another study (Table 3).^27^

**TABLE 3:** Assessment of cognitive function of the studies.

Author	Year	Location	Mean age	Sample size	Type of epilepsy	Study type	Mean duration of follow‐up	Mean duration of disease	Seizure frequency	Outcomes of VFT
(Kawai et al.^21^)	2017	Japan	23	362	tonic–clonic seizure	prospective cohort	36 months	15.6 years	106/week	There were no substantial changes in cognitive function
(Bamogaddam et al.^24^)	2020	Saudi Arabia	20.33	18	Myoclonic, GTC	Retrospective cohort	36 months	19.61 years	Unknown	Cognitive impairment improved (77.8%)
(Hallböök et al.^27^)	2005	Sweden	11	15	complex partial	prospective longitudinal study	9 months	8.5 years	51/month	No differences in cognitive functioning before and after VNS

## 4 DISCUSSION

This systematic review encompasses 11 clinical studies that investigated the impact of vagus nerve stimulation (VNS) on the management of seizures, quality of life, and cognitive function in individuals with drug‐resistant epilepsy. Each of the patients had a vagus nerve stimulator placed in their left neck after being diagnosed with refractory epilepsy. According to the findings, practically all refractory epilepsy patients had a significant decrease in seizure frequency and a considerable improvement in quality of life with VNS compared to before therapy. This conclusion is in line with published systematic reviews and meta‐analyses, and it confirms once more the effectiveness of VNS in reducing seizures, enhancing anxiety or depression, and enhancing quality of life.^30^, ^31^

Among drug‐resistant epilepsy patients in our review, the pooled ≥50% seizure reduction rate was 56.94% ranged from 48.90% to 83.00%. Similarly, a different study demonstrates that in DRE of any cause, seizure reduction rates of at ≥50% were attained, ranging from 37.6% to 64.8%.^32^ A 55% reduction was achieved in another pooled analysis involving 481 responders with all etiologies.^5^ These results imply that VNS therapy has a positive effect on DRE patients.

The evidence consistently points to a growing benefit over time, even though it is still unclear what factors will determine how well VNS therapy works. While research results vary, it appears that the duration, kind, and origin of epilepsy all influence the likelihood that VNS will be successful.

In addition to enhancing seizure control, VNS has improved patients' quality of life who have drug‐resistant epilepsy (Table 2). There may be advantages to mood and cognitive symptoms as well (Table 3), although further research is required on these, this finding is also similar with another study done in London, United Kingdom.^33^

Patients must regularly be evaluated during follow‐up for potential long‐term adverse effects. There are general recommendations on interventions and circumstances that may affect the proper functioning of VNS, but there are no particular guidelines for managing patients with VNS.

## 5 CONCLUSION

Our review had a mean response rate of 56.94%. The studies had response rates ranging from 48.90% to 83.00%. The review's conclusions suggest that VNS use may be advantageous for those with drug‐resistant epilepsy. This research also provides an overview of clinical trials pertaining to the use of VNS in patients with drug‐resistant epilepsy; hence, it could potentially function as a reference for future studies of a similar nature.

### 5.1 Limitations

One of our review's drawbacks is that we only included a limited sample of 11 studies to pool the results of treatment response, quality of life, and cognitive function, as well as seizure frequency. This meant that meta‐regression was not possible. A further limitation of our evaluation was the clinical heterogeneity across the studies (different treatment modalities, patients' underlying diseases, experience of the various centers with VNS, treatment duration, and stimulation parameters), as well as the moderate‐to‐high risk of bias in some of the studies.

## AUTHOR CONTRIBUTIONS

All authors agreed to be accountable for all aspects of the work and made significant contributions to the reported work, whether in terms of conception, study design, implementation, data extraction, and interpretation, or all of these areas combined. They also agreed on the journal to which the article was submitted, and they were involved in drafting, revising, or critically reviewing the article before giving final approval for it to be published.

## CONFLICT OF INTEREST STATEMENT

Neither of the authors has any conflict of interest to disclose. This paper was our original work and has not yet been published. The authors of this work assume full responsibility for its preparation and content, and they all attest to the absence of any conflicts of interest.

## ETHICS STATEMENT

We confirm that we have read the Journal's position on issues involved in ethical publication and affirm that this report is consistent with those guidelines.

## Data Availability

The data that support the findings of this study are available on request from the corresponding author. The data are not publicly available due to privacy or ethical restrictions.
